# Surgical outcomes and long-term survival of laparoscopic distal gastrectomy at high-volume centers in Korea and China: a two-centered retrospective analysis

**DOI:** 10.1007/s00595-024-02931-w

**Published:** 2024-11-19

**Authors:** Sen Wang, Ji-Hyeon Park, Qingya Li, Yikai Shen, Jee-Sun Kim, Do-Joong Park, Seong-Ho Kong, Haisheng Fang, Hye-Seung Lee, Linjun Wang, Diancai Zhang, Hao Xu, Hyuk-Joon Lee, Zekuan Xu, Han-Kwang Yang

**Affiliations:** 1 The Department of General Surgery, The First Affiliated Hospital of Nanjing Medical University, Nanjing, China; 2 Gastric Cancer Center, The First Affiliated Hospital of Nanjing Medical University, Nanjing, China; 3 Division of Gastrointestinal Surgery, Department of Surgery, Seoul National University Hospital, Seoul National University College of Medicine, Seoul, Korea; 4 Gastric Cancer Center, Seoul National University Hospital, Seoul National University College of Medicine, Seoul, Korea; 5 The Department of Surgery, Gachon University College of Medicine, Gachon University Gil Medical Center, Incheon, Korea; 6 The Department of Pathology, The First Affiliated Hospital of Nanjing Medical University, Nanjing, China; 7 The Department of Pathology, Seoul National University Hospital, Seoul, Korea

**Keywords:** Laparoscopic-assisted distal gastrectomy, Totally laparoscopic distal gastrectomy, Complications, Lymph nodes metastasis

## Abstract

**Purpose:**

Laparoscopic distal gastrectomy is now widely used in East Asia and worldwide with different preferences and outcomes. This study aimed to compare the short- and long-term outcomes and preferences between two high-volume gastric cancer centers in Korea and China.

**Methods:**

Patients who underwent laparoscopic-assisted distal gastrectomy (LADG) and totally laparoscopic distal gastrectomy (TLDG) for gastric cancer from Seoul National University Hospital (SNUH) and the First Affiliated Hospital of Nanjing Medical University (NMUH) from 2017 to 2020 were enrolled in this study.

**Results:**

A total of 1166 SNUH cases and 847 NMUH cases enrolled in this study. The overall complication rate of SNUH (14.49%) did not differ from that of NMUH after LADG or TLDG (12.28%). The anastomosis-related complications rate (2.74%) did not show a significant difference with that of NMUH (2.01%) either. The median postoperative hospital stay for SNUH (7,(7,10)) was shorter than that for NMUH (8,(7,9)). The 5-year overall survival (OS) rate of SNUH was not significantly different from that of NMUH.

**Conclusion:**

There was no significant difference in the overall complication rate, anastomosis-related complication rate, resected lymph nodes, and 5- year overall survival rate between SNUH and NMUH except for the postoperative stay. Both the LADG and TLDG achieved satisfactory short- and long-term outcomes when performed by surgeons with adequate experience.

**Supplementary Information:**

The online version contains supplementary material available at 10.1007/s00595-024-02931-w.

## Introduction

Gastric cancer (GC) is one of the most prevalent malignant tumors worldwide, and it is now ranked No.5 according to the latest statistics, with over one million patients diagnosed with GC every year. According to global cancer statistics, GC is more prevalent in East Asia, including China, Japan, Korea, and other countries [1]. China accounts for half of the 1.08 million new cases per year, while GC holds the highest incidence among all cancers in Korea [2]. Thus, the treatment of GC is relatively more common in East Asian countries, especially in high-volume centers.

While comprehensive treatments for GC, such as chemotherapy and immunotherapy, are increasingly adopted, surgery remains the primary radical treatment for GC [3]. Laparoscopic gastrectomy is now widely used in experienced centers and it is considered to be a safe and feasible method compared with traditional open surgery, especially for distal gastrectomy. With more evidence, laparoscopic distal gastrectomy has shown non-inferior effects on survival and complications compared to open gastrectomy. However, variations in surgical methods, reconstruction, and lymph node dissection persist among surgeons and centers. To date, no studies have directly compared the preferences and surgical outcomes between large gastric cancer centers in Korea and China, which may hinder our understanding of the different epidemic features and clinical preferences in different countries.

With regard to the prognosis, the results of recent large RCTs have shown that the survival of patients who underwent laparoscopic gastrectomy and open gastrectomy was comparable and they also showed demonstrated acceptable results [4–6]. However, as mentioned previously, no direct comparison of long-term survival has been conducted between Korean and Chinese large gastric cancer centers to demonstrate the latest prognosis data.

This study included Seoul National University Hospital (SNUH) from Korea and the First Affiliated Hospital of Nanjing Medical University (NMUH) from China, and aimed to compare the preference, surgical outcomes, and prognosis of LDG. The annual number of gastrectomies at these two renowned centers has reached over 800 with a long history of laparoscopic gastrectomy application, which could represent a general picture of the two countries to some extent. This would help directly resolve the divided opinions among different methods and characteristics and help understand whether the variance of laparoscopic methods may affect the outcomes.

## Methods

Consecutive cases of laparoscopic distal gastrectomy for gastric cancer performed between 2017 and 2020 were enrolled at SNUH and NMUH in this retrospective study. The pathological confirmation of stomach adenocarcinoma in all patients was based on the 8^th^ AJCC TNM staging system. We included Billroth I, Billroth II, Roux-en-Y, and Uncut Roux-en-Y anastomoses with either a totally laparoscopic or laparoscopic-assisted method. Note that B-II included all cases with or without Braun anastomosis. The complication criteria were normalized using the same standards. Complications included wound, fluid collection, intra-abdominal bleeding, stenosis, motility disorder, gastrointestinal (GI) leakage, gastrointestinal (GI) bleeding, other fistulas, ischemia, reflux, pulmonary complications, urinary complications, renal complications, hepatobiliary complications, and other gastrointestinal complications. The data were prospectively recorded by residents and statisticians and then reported to all surgical staff every week for verification and analysis at both centers. Postoperative complications that occurred within 30 days after surgery were assessed according to the Clavien–Dindo classification. The exclusion criteria included metachronous and synchronous malignancies and the combined resection of other main organs. Patients who underwent palliative surgeries were excluded from the database. Single-port laparoscopic gastrectomy was also excluded because not all surgeons performed single-port laparoscopy in both centers. Patients with conversion to open surgery were not included in this study. The R1 and R2 resections were excluded. We noticed that in some methods, the number of cases was quite small, which might have caused some bias in the analysis. To eliminate any potential bias, if the number of cases was less than ten, analysis was not performed. Since the distribution of postoperative stay was not a normal disruption, we used the median to indicate the postoperative hospital stay analysis. To compare the 5-year overall survival rate, we selected patients from 2017 to 2018 that fulfilled a follow-up time of 5 years.

The mapping procedure for the specimen between the two centers followed the same protocols. Pathological staging was delineated according to the 8^th^ edition of the AJCC Staging System, which was verified by pathologists from both centers. All sections were evaluated for the deepest invasion to ensure accurate T staging. For advanced gastric cancer, pathologists retrieved the suspected portion that was the most invasive in depth by the naked eye and then made sections with a diameter of 2 cm, centering on the deepest portion. The statistical software program SPSS 26 was used for the analysis. Continuous variables are described as the mean ± SD or median (25%,75%), and categorized variables are summarized by frequency (n) and proportion (%). Student’s *t* test and the Chi-square test were used for rate or proportion comparisons. Logistic regression was also used for the multivariate analysis. Statistical significance was set at *p* < 0.05. This study was approved by the ethical committees (IRB) of SNUH and NMUH. The institutional review board (IRB) number was 2110-036-1260. Written consent was obtained from all patients at the SNUH and NMUH.

## Results

### Overview and preferences of SNUH and NMUH

After screening patients for inclusion and exclusion criteria (Supplementary Fig. 1), 1166 cases including 780 males and 386 females from SNUH and 847 cases including 552 males and 295 females from NMUH were enrolled in this study (Supplementary Table 1). Their ages ranged from 28 to 88 years in SNUH and from 26 to 87 years in NMUH. In the SNUH group, 315 and 851 patients underwent LADG and TLDG, respectively. In the NMUH group, only 90 underwent LADG, while 757 underwent TLDG. As shown in Table S1, there were significant differences in age (*p* = 0.001), pathological T stage (pT) (*p* < 0.001), pathological N stage (pN) (*p* < 0.001), LADG methods (*p* < 0.001), TLDG methods (*p* < 0.001), and tumor size (*p* < 0.001) between SNUH and NMUH, except for sex (*p* = 0.420) and tumor location (*p* = 0.460). Notably, 71.78% of the enrolled GC patients were diagnosed at the pT1 stage in SNUH and 51.83% at the pT1 stage in NMUH. This indicated some differences in the preferences and baseline parameters between SNUH and NMUH.

At SNUH, we found that LADG RY (*n* = 2, 0.17%), LADG Uncut (*n* = 0, 0%), TLDG RY (*n* = 23, 1.97%), and TLDG Uncut (*n* = 20, 1.71%) were rarely performed. B-I (460, 39.45%) and B-II (*n* = 661, 56.69%) were performed more frequently in the SNUH group than RY and Uncut . In the NMUH group, TLDG B-II (*n* = 464, 54.78%) and TLDG Uncut (*n* = 209, 24.68%) were performed, thus indicating a preference for the TLDG method. The LADG method was also performed, but similar to the LADG RY/Uncut to SNUH, the total number of LADG cases was 90 (10.62%) in NMUH, especially in LADG B-I (*n* = 1, 0.12%). Irrespective of LADG or TLDG, B-I was seldom performed in NMUH.

Pathological T1 stage gastric cancer was more prevalent in SNUH (71.78%) in all laparoscopic distal gastrectomy cases, whereas in NMUH, only half of the patients were at the pT1 stage (51.83%). Along with more early pT stage cases in SNUH, For pT1 cases, we found 76.8% was pN0 at SNUH and 57.14% at NMUH.

### The overall complication rate did not differ between SNUH and NMUH

The number of patients with overall complications from both centers is shown in Table 1. No mortality occurred in any of the patients in our study. As shown in Table 1, SNUH and NMUH had overall LADG complication rates of 17.77% and 18.89%, respectively, with no significant difference (*p* = 0.809) in the LADG subgroup. The only available comparison showed no significant difference in the overall complication rate between the SNUH and LADG groups (*p* = 0.851).

**Table 1:** The overall information of complication rates of SNUH and NMUH

2017–2020		SNUH			NMUH			
Anastomosis		Cases	Complication cases	Complication rate	Cases	Complication cases	Complication rate	*p*-value
LADG	B-I	183	36	19.67%	1	1	–	–
	B-II	130	20	15.38%	36	6	16.67%	0.851
	RY	2	0	–	39	7	17.951%	–
	uncut	0	0	–	14	3	21.42%	–
	Total	315	56	17.77%	90	17	18.89%	0.809
TLDG	B-I	277	32	11.55%	7	1	14.29%	–
	B-II	531	74	13.94%	464	62	13.36%	0.853
	RY	23	5	21.74%	77	7	9.09%	0.203
	uncut	20	2	10.00%	209	17	8.13%	> 0.999
	Total	851	113	13.28%	757	87	11.49%	0.279
Total		1166	169	14.49%	847	104	12.28%	0.152

In the TLDG subgroup, the overall complication rate of TLDG cases from SNUH was 13.28%, which did not differ from the 11.49% for NMUH (*p* = 0.279). The B-I analysis could not be completed because of the lack of data. The overall complication rates of TLDG B-II (*p* = 0.853), RY (*p* = 0.203), and uncut (*p* > 0.999) analyses did not show any significant differences between SNUH and NMUH.

Finally, we combined all laparoscopic distal gastrectomy cases for comparison purposes between the two centers, which was 14.49% for SNUH versus 12.28% for NMUH (*p* = 0.152). These outcomes aligned with our expectations as they did not yield significantly different results. Additionally, we conducted a multivariate analysis of variance to assess whether factors such as age, sex, and cancer stage would impact the outcomes. However, the analysis yielded negative results (Supplementary Table 2), thus indicating that these factors did not influence our results.

### SNUH and NMUH did not differ regarding the anastomosis-related complication rates

Gastrointestinal anastomosis is one of the most important procedures performed during laparoscopic distal gastrectomy. As a result, we consider the anastomosis-related complications to have a closer correlation to the quality of laparoscopic surgery. The most serious complications associated with anastomosis are GI leakage and GI bleeding. Therefore, we categorized these as anastomosis-related complications. We conducted the same analysis as previously described for the overall complications (Table 2). The anastomosis-related complication of all LADG cases from SNUH (2.54%) and NMUH (5.55%) did not demonstrate a significant difference (*p* = 0.386), as well as the LADG B-II analysis (*p* = 0.206). In the TLDG analysis, B-II (*p* = 0.324), RY(*p* = 0.548) and Uncut (*p* = 0.259) analysis confirmed no difference between SNUH and NMUH again regarding anastomosis-related complications. Among all TLDG cases, 2.82% from SNUH and 1.59% from NMUH demonstrated negative results (*p* = 0.095). In general, when combining LADG and TLDG cases, there was no statistical difference between SNUH (2.74%) and NMUH (2.01%) for anastomosis-related complications (*p* = 0.251). We performed a multivariate analysis of variance again to detect if other factors would influence the outcomes, and the findings remained negative (Supplementary Table 3).

**Table 2:** The overall information of anastomosis-related complication rates of SNUH and NMUH

		SNUH			NMUH			
Anastomosis		Cases	Complication cases	Complication rate	Cases	Complication cases	Complication rate	*p*-value
LADG	B-I	183	6	3.28%	1	1	–	–
	B-II	130	2	1.54%	36	2	5.55%	0.206
	RY	2	0	–	39	1	2.56%	–
	uncut	0	0	–	14	1	7.14%	–
	Total	315	8	2.54%	90	5	5.55%	0.386
TLDG	B-I	277	8	2.88%	7	0	–	–
	B-II	531	14	2.64%	464	8	1.72%	0.324
	RY	23	1	4.35%	77	2	2.60%	0.548
	uncut	20	1	5.00%	209	2	0.96%	0.259
	Total	851	24	2.82%	757	12	1.59%	0.095
Total		1166	32	2.74%	847	17	2.01%	0.251

### Postoperative hospital stay of SNUH was shorter than that of NMUH

The detailed postoperative data are listed in Table 3. We have found that postoperative time of all LADG cases from SNUH (8(7,11))was statistically shorter than that of NMUH (9(8,10)) (*p* = 0.0163) in the LADG subgroup.The comparison of the postoperative time after LADG B-II did not show any positive results between SNUH and NMUH (*p* = 0.4095) while other comparisons were not available.

**Table 3:** The overall information regarding the length of postoperative stay for SNUH and NMUH

		SNUH		NMUH		
Anastomosis		Cases	Postoperative stay(d)	Cases	Postoperative stay(d)	*p*-value
LADG	B-I	183	8 (7, 11)	1	–	–
	B-II	130	8 (7, 9)	36	9 (7, 10.5)	0.4095
	RY	2	–	39	9 (8, 10)	–
	uncut	0	–	14	8 (8, 9)	–
	Total	315	8 (7, 11)	90	9 (8, 10)	0.0163
TLDG	B-I	277	7 (6, 9)	7	8 (8, 9.5)	–
	B-II	531	7 (7, 9)	464	7 (7, 9)	0.4094
	RY	23	8.5 (6.75, 12)	77	7 (7, 8)	0.9816
	uncut	20	7 (6, 7.5)	209	7 (7, 9)	0.4035
	Total	851	7 (6, 9)	757	7 (7, 9)	0.0017
Total		1166	7 (7, 10)	874	8 (7, 9)	0.0001

Consistent with the LADG sub-analysis, although B-II (*p* = 0.4094), RY (*p* = 0.9816), and Uncut (*p* = 0.4035) sub-analyses demonstrated negative results, all TLDG cases from SNUH (7(6,9)) had a shorter postoperative stay than those with NMUH(8,(7,9)) (*p* = 0.0017). Taken together, SNUH had a shorter postoperative hospital stay (7(7,10) days) than NMUH (8(7,9) days) (*p* = 0.0001). A multivariate analysis of variance confirmed that other factors did not influence the outcomes (Supplementary Table 4).

### Resected lymph nodes demonstrated no significant difference between SNUH and NMUH

We then analyzed the number of resected lymph nodes using different methods (Supplementary Table 5). The number of resected lymph nodes was confirmed by pathologists from both centers. Since D2 lymphadenectomy was performed in all cases of NMUH, D1 + lymphadenectomy was performed in early cases, and D2 lymphadenectomy was performed in advanced cases in SNUH, we selected all cases from NMUH and only D2 cases from SNUH. Based on these criteria, we included 187 LADG and 311 TLDG cases from SNUH. As previously reported, resected lymph nodes after B-II did not show a significant difference (*p* = 0.9718) between both centers, and the same situation emerged in the LADG subgroup compared with 37.43 ± 15.25 in SNUH and 39.67 ± 10.31 in NMUH (*p* = 0.2038). In the TLDG comparison, B-II (*p* = 0.3738), RY (*p* = 0.3263), and Uncut (*p* = 0.4710) showed similar results. Although resected LNs in SNUH with 40.92 ± 15.18, significantly fewer than 42.81 ± 11.23 of NMUH (*p* = 0.0263); however, after combining LADG and TLDG, no difference in resected LNs emerged between the two centers. Both centers retrieved more than 40 lymph nodes, which is much more than the number that the NCCN guidelines required.

### The lymph node metastasis rate was significantly higher in NMUH than in SNUH

Since the average number of retrieved LNs exceeded 40, this could result in some interesting facts. We then analyzed the distribution of LNs at different T stages (Table 4). Comparing the resected LNs between the two centers based on the pT stage, we found that the LNs of pT1a (*p* = 0.0002), pT1b (*p* = 0.0004), and pT2 (*p* = 0.0446)from SUNH were significantly fewer than those of NMUH, but no difference was observed in pT3 and pT4a stage tumors between SNUN and NMUH. This is expected because all cases of NMUH were performed with D2 dissection, but early cases of SNUH may be performed with D1 + dissection. Generally, the resected number of LNs is approximately 40, which is adequate for the pathological analysis.

**Table 4:** The resected LNs and LNM rate of the tumors in NMUH and SNUH

pT stage	SNUH				NMUH				*p*-value (Resected LN)	*p*-value (Metastasis rate)
	Cases	Resected LN	Metastasis cases	Metastasis rate	Cases	Resected LN	Metastasis cases	Metastasis rate		
T1a	418	37.15 ± 14.85	6	1.46%	235	41.05 ± 9.37	22	9.36%	0.0002	< 0.001
T1b	419	37.39 ± 14.80	85	20.29%	204	41.67 ± 10.37	64	31.37%	0.0004	0.002
T2	147	39.13 ± 15.22	71	48.30%	107	42.71 ± 10.39	61	57.01%	0.0446	0.170
T3	103	41.24 ± 15.97	67	65.05%	178	42.31 ± 9.77	134	75.28%	0.5008	0.067
T4a	55	41.07 ± 13.42	39	70.91%	92	42.01 ± 9.23	79	85.87%	0.6862	0.027
T4b	1	31	1	100%	2	58.5 ± 20.51	2	100%	/	/

We then calculated the lymph nodes metastasis (LNM) rate for each T stage GC, as listed in Table 4. In SNUH, the LNM rate of pT1a, pT1b, pT2, pT3, pT4a and T4b were 1.46%, 20.29%, 48.30%, 65.05%, 70.91% and 100%, respectively, while LN metastasis rate in NMUH of pT1a, pT1b, pT2, pT3, pT4a and pT4b were 9.36%, 31.37%, 51.37%, 75.38%, 85.87% and 100%. Interestingly, the LNM rate in NMUH was significantly higher than that in SNUH (nearly 10% higher at almost every stage). Statistical significance was observed for the pT1a (*p* < 0.001), pT1b (*p* = 0.002), and pT4a (*p* = 0.0027) stages.

We then analyzed the difference in tumor sizes of both centers, and the inspection of tumor size might influence LNM (Supplementary Fig. 2). More interestingly, the tumor size of pT1a (*p* = 0.0001), pT1b (*p* = 0.0001), and combined (*p* = 0.0001) in SNUH was significantly larger than that in NMUH and regarding pT4a there was no significant difference (*p* = 0.0758), thus indicating that size did not play a role in the LNM rate variance.

### No significant difference in the 5-year OS between SNUH and NMUH

**Fig. 1:**
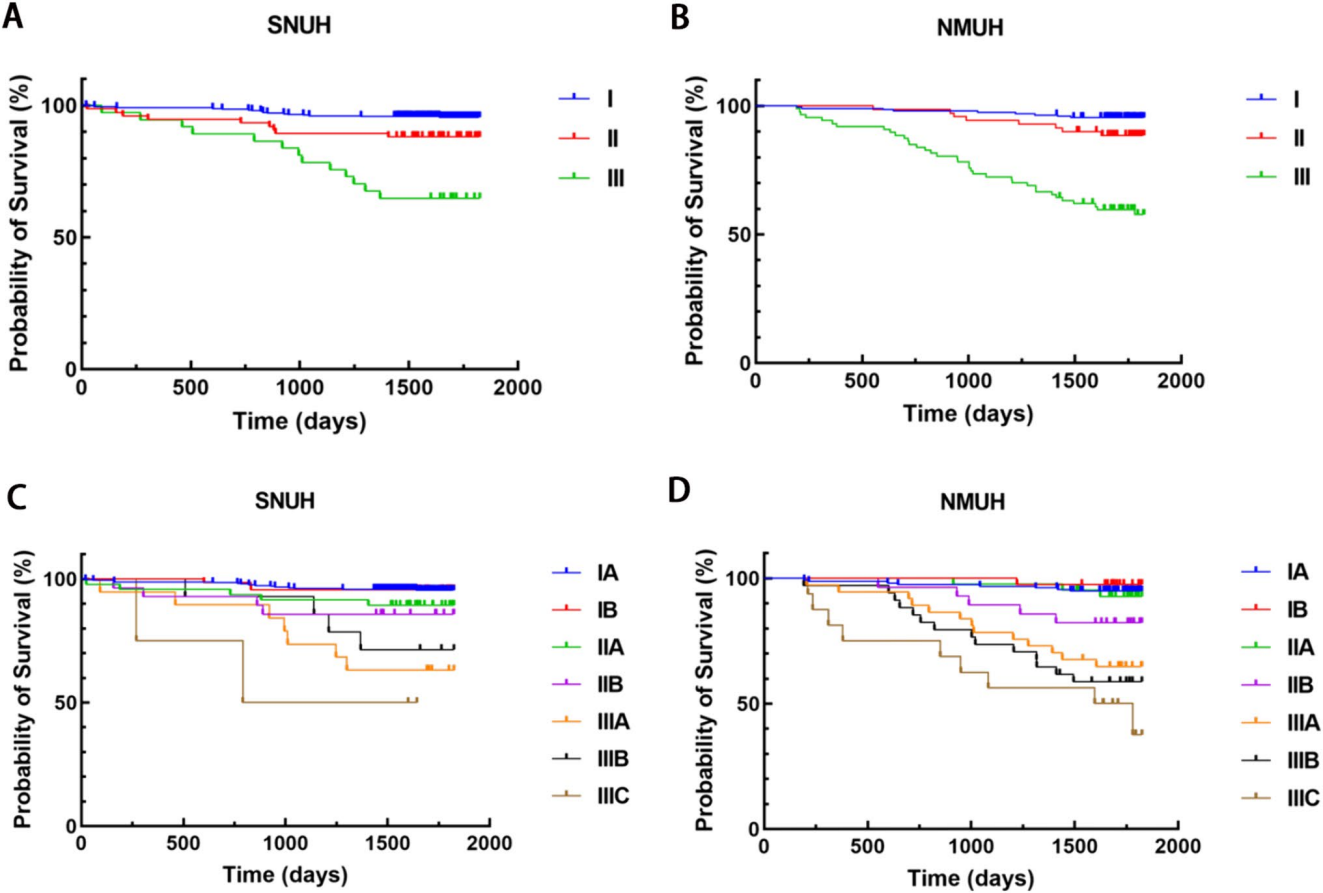
5-year overall survival for different stages in SNUH and NMUH

We analyzed the overall survival time of patients enrolled from the SNUH and NMUH groups. Combining all enrolled patients, SNUH exhibited a 5-year overall survival rate of 92.22%, while that of NMUH was 84.94% (χ^2^ = 11.534, *p* = 0.001). Subsequently, we further compared the 5-year OS of SNUH and NMUH patients based on stages I, II, and III, which revealed no significant difference between the two centers (Fig. 1, Table 5). Similarly, no statistically significant differences were observed between the two centers when further comparisons were made based on the specific TNM stages (Fig. 1, Table 5). In addition, a risk analysis of the prognosis of the two centers was conducted. A multivariate Cox analysis demonstrated that more advanced T and N stages would affect the prognosis, as expected. However, neither different centers nor laparoscopic methods influenced prognosis according to the analysis (Supplementary Table 6).

**Table 5:** No significant differences in the 5-year prognosis were observed between SNUH and NMUH

2017–2018	SNUH			NMUH			*p*-value
TNM stage	Cases	Death cases	5-year OS rate	Cases	Death cases	5-year OS rate	
I	402	18	95.52%	195	9	95.38%	0.939
II	75	9	88.00%	70	8	88.57%	0.915
III	37	13	64.86%	87	36	58.62%	0.515
I_A_	335	15	95.52%	156	8	94.87%	0.751
I_B_	67	3	95.52%	39	1	97.44%	> 0.999
II_A_	47	5	89.36%	42	3	92.86%	0.717
II_B_	28	4	85.71%	28	5	82.14%	> 0.999
III_A_	19	7	63.16%	37	13	64.86%	0.900
III_B_	14	4	71.43%	34	14	58.82%	0.412
III_C_	4	2	50.00%	16	9	43.75%	> 0.999

## Discussion

This study investigated the distribution of gastric cancer, surgical outcomes, and prognosis between two high-volume gastric cancer centers in Korea and China. A previous study compared the morbidity and mortality of patients who underwent gastrectomy between a Chinese and an American center, suggesting no difference in prognosis [7]. This is the first study to display the variance in preference and baseline characteristics, which also directly compared the outcomes of gastric cancer between Korea and China. First, we noticed a difference in the pathological stages between SNUH and NMUH. As shown in Table 1, early cases were much more prevalent in SNUH than in NMUH, with more pN0 cases in SNUH than in NMUH. This could be partially explained by better screening work in Korea and the comparatively smaller population of Korean patients diagnosed with GC earlier. However, we observed a relatively higher proportion of pT1 stage cases in NMUH than expected, while considering the prevalence of more advanced cases in China. We focused on the TLDG and LADG between SNUH and NMUH. LADG B-I and B-II are the most commonly performed anastomoses in SNUH, while only a few cases have been performed with LADG in NMUH compared to TLDG. This variance may be attributed to the higher familiarity with circular stapler application in SNUH. It resembles open surgery, and a circular stapler is perceived as safe, with a lower risk of stenosis.. Additionally, esophagojejunostomy in totally laparoscopic total gastrectomy is usually performed using a circular stapler in SNUH. While some publications suggest that both linear and circular methods are safe and feasible, others suggest that the linear stapler approach may result in shorter operation times [8, 9]. LADG is not commonly performed in NMUH. Most cases were performed with intracorporeal gastrojejunostomy using a linear stapler, which differs from SNUH. In addition, regarding LADG versus TLDG, another concern is the ability to determine a proper proximal margin. By LADG, surgeons can easily confirm the proper proximal margin through minilaparotomy and control any spealing of gastric content during resection and anastomosis, especially in advanced cancer, which is one of the reasons why LADG was more frequently applied in SNUH than in SNUH [10, 11]. On the other hand, intraoperative gastroscopic examinations or preoperative endoscopic clips might help determine the proper proximal margin [12]. To date, large RCTs have not yet confirmed any significant difference between TLDG and LADG, so now it is still more about the preference for choosing linear or circular staplers. KLASS-07 results may provide further evidence for this issue [13, 14].

Regarding B-I, B-II, RY, and Uncut, we found that B-I and B-II were the major anastomoses in SNUH, while B-II and Uncut were the leading anastomoses in NMUH. Owing to the similarity of B-II and Uncut, we could generally consider B-II and Uncut as the same type with the same gastrojejunostomy. As mentioned above, surgeons from NMUH are more comfortable with linear staplers intracorporeally, which naturally led to more TLDG B-II and Uncut cases. Under these circumstances, afferent/efferent loop syndrome and reflux may occur after B-II surgery. Regarding B-I, the similar structure of the natural digestive tract may cause the least side effects and complications in patients. However, the high tension of the gastroduodenostomy may cause leakage, which requires the duodenal stump to be dissected long enough for anastomosis, which is quite demanding in terms of the skills and location of the tumor. Based on the China Gastrointestinal Cancer Surgery Union, B-I is not the first option for anastomosis in laparoscopic distal gastrectomy in China [15]. Regarding LADG or TLDG, even though the preference of anastomosis varied, no significant difference on LADG overall complication rate, TLDG overall complication rate and overall complication rates of all cases between SNUH and NMUH, as well as anastomosis-related complication comparisons, thus indicating that the surgical outcomes were not biased according to experience. This showed that both TLDG and LADG were safe and stable for complications at different centers. In fact, the overall complication rates after LADG and TLDG were satisfactory. The overall complication rate after laparoscopic distal gastrectomy in KLASS-02 was 15.7%, while the JCOG0912 trials have suggested higher overall complication rates, and our results were therefore comparable to those of previous studies (SNUH: 14.49%, NMUH:12.28%) [5, 16, 17]. In this study, despite the variances in laparoscopic methods, no significant difference in complications was observed, thus indicating that these factors did not affect the short-term results. With adequate experience, acceptable complication rates can be achieved after each type of anastomosis.

Regarding the postoperative stay, SNUH had shorter postoperative days than NMUH regardless of LADG, TLDG, or all cases. However, this is considered to be a comparatively weak parameter, as different hospitals have different discharge policies depending on the surgeon’s decision, but this also partially demonstrates the postoperative management and recovery speed of patients. The difference in the median postoperative day after LADG between SNUH and NMUH is reasonable because there were fewer cases of LADG experience in NMUH. We analyzed the data again and noticed that the postoperative median time of TLDG was the same between SNUH and NMUH, meaning that most patients were discharged on POD7 at both centers. Meanwhile, 20% of SNUH patients were discharged less than POD7, including 5 or 6 days, while only 11% of patients were discharged less than POD7 in the NMUH group. Only nine patients were discharged at POD5 in the NMUH group, while 114 were discharged in the SNUH group. This suggested that ERAS was better applied in SNUH, which resulted in a faster recovery. This could partially explain why the median postoperative day was the same, but the 25^th^ percentile of postoperative time was lower in the SNUH group. This discrepancy was statistically significant, although the complication rates were similar. Despite the statistical difference, the clinical difference may not be substantial, and this outcome may not reflect the actual surgical outcomes between the two centers.

Regarding lymph nodes based on T stage, in NMUH, the average number of lymph nodes at different stages was not significantly different because all cases was performed in D2 lymphadenectomy. In SNUH, early cases underwent D1 + lymphadenectomy. The number of resected LNs in pT1 cases in SNUH was slightly lower than that in NMUH cases. However, these differences were relatively small, and the number of resected LNs for each type of surgery was approximately 40, thereby surpassing the requirements outlined in the guidelines..

Interestingly, we discovered significant differences in the proportion of patients with positive lymph node metastasis between the two centers. Previous studies have reported varying rates of lymph node metastasis in patients with pT1a gastric cancer. For instance, in a study conducted in SNUH from 2008 to 2012, the mean number of retrieved LNs was 35.5 among 1003 pT1a GC, with 18 (1.8%) of them having LN metastasis [18]. Milhomen reported a 3.96% (3/76) LNM rate in T1a tumors [19] and Bausy suggested a 4.95% rate for pT1a tumors [20]. According to the Chinese GI Cancer Surgery Union, the pT1b LNM rate is 19.3%, which is comparable to that of SNUH (15). However, some studies have reported even higher rates of lymph node metastasis in both pT1a and pT1b tumors. Ikoma et al. suggested a 10% LNM rate for pT1a tumors [21]. After analyzing over 40,000 patients from the SEER database, Pokala et al. reported a gradually increasing lymph node metastasis rate of up to 20% for low-grade T1a tumors as the tumor size increased from 0 to 1 cm to > 4 cm [22]. Similarly, Bausy et al. and Ikoma et al. reported LNM rates of 31.9% and 34%, respectively, in pT1b tumors [20, 21]. These findings suggest that the LNM rates of pT1a and pT1b may not be as low as generally considered. Interestingly, tumor size did not account for the higher lymph node metastasis rate observed in the NMUH group. Choi et al. suggested that the LNM rate in pT1a tumors varies among races, with the highest rates in whites and blacks, and the lowest in Asians [23]. This prompted us to consider whether there could be racial differences between Koreans and Chinese. Our results also suggest that the higher LNM rate in China may not be by chance, but instead may rather be a common phenomenon.

The long-term prognosis is also a very important factor in determining the quality of treatment at gastric cancer centers. We found that the 5-year overall survival rate of enrolled patients with SNUH was superior to NMUH (92.22% vs. 84.94%, *χ*^2^ = 11.534, *p* = 0.001), which was probably due to the higher number of early cases in Korea. However, after staging, there was no statistically significant difference in the 5-year survival between the two centers. The prognosis of each stage was very similar between the two centers, thus indicating high-quality treatment and standards. For stage IIIB-C patients, the prognosis of SNUH seemed better than that of NMUH, but the difference was not statistically significant. This could be due to the limited number of cases of IIIB-IIIC. According to CLASS-01, the 5-year overall survival (OS) rate of patients with locally advanced gastric cancer was 72.6% in the laparoscopic distal gastrectomy (LDG) group and 76.3% in the open distal gastrectomy (ODG) group (*p* = 0.19), while gastric cancer mortality (*p* = 0.34) and deaths from other causes (*p* = 0.42) did not differ significantly between the groups [24]. For patients with stage I gastric cancer who underwent either laparoscopic or open surgery, the 5-year OS rate (94.2% vs. 93.3%, *p* = 0.64) and cancer-specific survival rate (97.1% vs. 97.2%, *p* = 0.91) did not show significant differences between the groups according to KLASS-01[25]. Generally, both centers demonstrated non-inferior and comparable results in the 5-year survival, thus indicating that different preferences or methods would not affect the prognosis, which was also confirmed by a multivariate analysis of the prognosis.

This study is associated with some limitations. Due to the lack of some anastomotic methods (such as LADG B-I in NMUH) in both centers, it may not be adequate for analysis in further studies and the results could be biased. As this was a retrospective study, some selection bias may have influenced the results. As a result, a large-scale international multicenter RCT is required for further analyses.

## Conclusion

There was no significant difference in the overall complication rate, anastomosis-related complication rate, resected lymph nodes, and 5-year overall survival rate between SNUH and NMUH, except for the length of the postoperative stay. Both the LADG and TLDG achieved satisfactory short- and long-term outcomes when performed by surgeons with adequate experience.

## Supplementary Information

Below is the link to the electronic supplementary material.

Supplementary file1 (DOCX 29 KB)

Supplementary file2 (DOCX 13647 KB)
