# Supplementary material for: Surgical outcomes and long-term survival of laparoscopic distal gastrectomy at high-volume centers in Korea and China: a two-centered retrospective analysis
Source: Surg Today. 2024 Nov 19;55(1):52–61. doi: 10.1007/s00595-024-02931-w (PMC11717828; doi:10.1007/s00595-024-02931-w)
Supplement: Supplementary file 1 — Supplementary file1 (DOCX 29 KB) [file 595_2024_2931_MOESM1_ESM.docx]

Supplementary Tables

Supplementary Table 1: Detailed information regarding the patients enrolled in this study from SNUH and NMUH.

|  | | SNUH | NMUH | *p*-value |
| --- | --- | --- | --- | --- |
| Number of patients | | 1166 | 847 |  |
| Age(y) | | 62.91±11.46 (28-88) | 60.05±10.94 (26-87) | 0.001 |
| Gender | M | 780(66.90%) | 552(65.17%) | 0.420 |
|  | F | 386(33.10%) | 295(53.93%) |  |
| Pathological  T stage | T1 | 837(71.78%) | 439(51.83%) | <0.001 |
|  | T2 | 147(12.61%) | 107(12.63%) |  |
|  | T3 | 103(8.83%) | 178(21.02%) |  |
|  | T4 | 56(4.80%) | 94(11.10%) |  |
|  | Other | 23(1.97%) | 29(3.42%) |  |
| Pathological  N stage | N0 | 896(76.84%) | 484(57.14%) | <0.001 |
|  | N1 | 129(11.06%) | 111(13.11%) |  |
|  | N2 | 89(7.63%) | 104(12.28%) |  |
|  | N3a | 38(3.26%) | 100(11.81%) |  |
|  | N3b | 14(1.20%) | 48(5.67%) |  |
| LADG | B-I | 183(15.70%) | 1(0.12%) | <0.001 |
|  | B-II | 130(11.15%) | 36(4.25%) |  |
|  | RY | 2(0.17%) | 39(4.60%) |  |
|  | Uncut | 0(0%) | 14(1.65%) |  |
| TLDG | B-I | 277(23.76%) | 7(0.83%) | <0.001 |
|  | B-II | 531(45.54%) | 464(54.78%) |  |
|  | RY | 23(1.97%) | 77(9.09%) |  |
|  | Uncut | 20(1.71%) | 209(24.68%) |  |
| Tumor location | Gastric antrum | 675(57.89%) | 469(55.37%) | 0.460 |
|  | Gastric angle | 301(25.81%) | 225(26.57%) |  |
|  | Others | 190(16.30%) | 153(18.06%) |  |
| Tumor size | ≤ 2cm | 309(26.50%) | 362(42.74%) | <0.001 |
|  | 2-5cm | 655(56.18%) | 372(43.92%) |  |
|  | ≥ 5cm | 202(17.32%) | 113(13.34%) |  |

Supplementary Table 2: Univariate and multivariate analyses of variance in the overall complications rates between SNUH and NMUH. (**p*<0.05).

| Variables | Category | Univariate analysis | Multivariate analysis |
| --- | --- | --- | --- |
|  |  | p | p |
| Gender |  | 0.172 | 0.567 |
| Age |  | 0.109 | 0.459 |
| T stage | T1 | 0.132 | / |
|  | T2 |  | 0.532 |
|  | T3 |  | 0.186 |
|  | T4 |  | 0.314 |
| N stage | N0 | 0.536 | / |
|  | N1 |  | 0.179 |
|  | N2 |  | 0.920 |
|  | N3a |  | 0.838 |
|  | N3b |  | 0.530 |
| Anastomoses | B-I | 0.196 | / |
|  | B-II |  | 0.691 |
|  | Roux-en-Y |  | 0.115 |
|  | Uncut Roux-en-Y |  | 0.750 |

Supplementary Table 3: Univariate and multivariate analyses of variance in the anastomosis-related complication rates between SNUH and NMUH. (**p*<0.05).

| Variables | Category | Univariate analysis | Multivariate analysis |
| --- | --- | --- | --- |
|  |  | p | p |
| Gender |  | 0.361 | 0.806 |
| Age |  | 0.181 | 0.386 |
| T stage | T1 | 0.341 | / |
|  | T2 |  | 0.328 |
|  | T3 |  | 0.065 |
|  | T4 |  | 0.140 |
| N stage | N0 | 0.852 | / |
|  | N1 |  | 0.889 |
|  | N2 |  | 0.898 |
|  | N3a |  | 0.282 |
|  | N3b |  | 0.526 |
| Anastomoses | B-I | 0.465 | / |
|  | B-II |  | 0.691 |
|  | Roux-en-Y |  | 0.115 |
|  | Uncut Roux-en-Y |  | 0.750 |

Supplementary Table 4: Univariate and multivariate analyses of variance in the length of postoperative hospital stays between SNUH and NMUH. (**p*<0.05).

| Variables | Category | Univariate analysis | Multivariate analysis |
| --- | --- | --- | --- |
|  |  | p | p |
| Gender |  | 0.216 | 0.633 |
| Age |  | 0.106 | 0.081 |
| T stage | T1 | 0.287 | / |
|  | T2 |  | 0.889 |
|  | T3 |  | 0.143 |
|  | T4 |  | 0.622 |
| N stage | N0 | 0.102 | / |
|  | N1 |  | 0.140 |
|  | N2 |  | 0.912 |
|  | N3a |  | 0.591 |
|  | N3b |  | 0.646 |
| Anastomoses | B-I | 0.243 | / |
|  | B-II |  | 0.919 |
|  | Roux-en-Y |  | 0.485 |
|  | Uncut Roux-en-Y |  | 0.739 |

Supplementary Table 5: The overall information regarding the resected lymph nodes of SNUH and NMUH.

|  | | SNUH | | NMUH | | *p*-value |
| --- | --- | --- | --- | --- | --- | --- |
| Anastomosis | | Cases | Resected LN | Cases | Resected LN |  |
| LADG | B-I | 55 | 41.44±16.89 | 1 | 28 | / |
|  | B-II | 130 | 41.45±17.10 | 36 | 41.97±8.64 | 0.9718 |
|  | RY | 2 | 69±28.28 | 39 | 38.36±11.45 | / |
|  | uncut | 0 | / | 14 | 38.21±10.53 | / |
|  | Total | 187 | 42.18±17.26 | 90 | 39.67±10.31 | 0.2038 |
| TLDG | B-I | 97 | 39.18±12.33 | 7 | 41.29±9.16 | / |
|  | B-II | 190 | 41.84±15.45 | 464 | 42.81±11.35 | 0.3738 |
|  | RY | 10 | 44.90±17.30 | 77 | 40.64±10.03 | 0.3263 |
|  | uncut | 14 | 40.93±20.19 | 209 | 43.64±11.38 | 0.4170 |
|  | Total | 311 | 41.06±14.84 | 757 | 42.81±11.23 | 0.0263 |
| Total | | 498 | 41.48±15.78 | 847 | 42.47±11.17 | 0.1838 |

Supplementary Table 6: Multivariate Cox proportional hazards survival analysis.

| Variables | Category | HR (95%CI) | Multivariate  P value |
| --- | --- | --- | --- |
| Hospital |  | 1.157 (0.707-1.893) | 0.562 |
| Gender |  | 0.989 (0.658-1.488) | 0.959 |
| Age |  | 1.065 (1.045-1.087) | ＜0.001 |
| Laparoscopy methods |  | 0.911 (0.546-1.518) | 0.720 |
| T stage | T1 | / | / |
|  | T2 | 0.874 (0.389-1.964) | 0.744 |
|  | T3 | 2.215 (1.208-4.061) | 0.010 |
|  | T4 | 4.051 (2.144-7.654) | ＜0.001 |
| N stage | N0 | / | / |
|  | N1 | 1.169 (0.558-2.449) | 0.679 |
|  | N2 | 3.021 (1.629-5.602) | ＜0.001 |
|  | N3a | 2.993 (1.544-5.804) | 0.001 |
|  | N3b | 7.770 (3.799-15.894) | ＜0.001 |
| Anastomoses | B-I |  | / |
|  | B-II | 1.087 (0.574-2.062) | 0.797 |
|  | Roux-en-Y | 1.036 (0.384-2.791) | 0.945 |
|  | Uncut Roux-en-Y | 0.958 (0.413-2.224) | 0.921 |
