# Supplementary material for: Surgical outcomes and long-term survival of laparoscopic distal gastrectomy at high-volume centers in Korea and China: a two-centered retrospective analysis
Source: Surg Today. 2024 Nov 19;55(1):52–61. doi: 10.1007/s00595-024-02931-w (PMC11717828; doi:10.1007/s00595-024-02931-w)
Supplement: Supplementary file 2 — Supplementary file2 (DOCX 13647 KB) [file 595_2024_2931_MOESM2_ESM.docx]

Supplementary Figures

Supplementary Figure 1: A chart flow of the screening procedures.


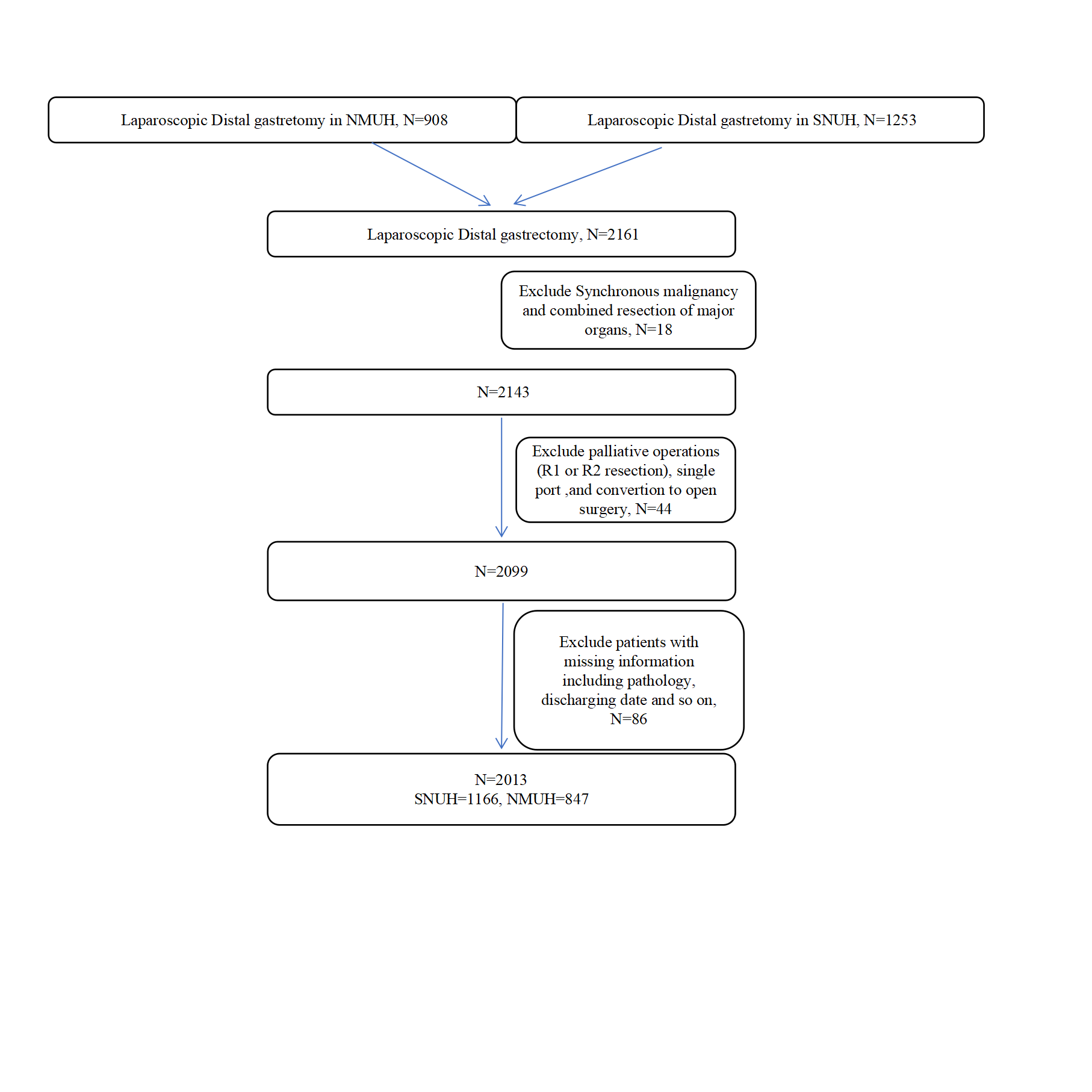


Supplementary Figure 2: An analysis of the tumor sizes of NMUH and SNUH.
